# *In vitro* and *in vivo* Effects of Lactate on Metabolism and Cytokine Production of Human Primary PBMCs and Monocytes

**DOI:** 10.3389/fimmu.2018.02564

**Published:** 2018-11-12

**Authors:** Jacqueline M. Ratter, Hanne M. M. Rooijackers, Guido J. Hooiveld, Anneke G. M. Hijmans, Bastiaan E. de Galan, Cees J. Tack, Rinke Stienstra

**Affiliations:** ^1^Department of Internal Medicine, Radboud University Medical Center, Nijmegen, Netherlands; ^2^Nutrition, Metabolism and Genomics Group, Division of Human Nutrition and Health, Wageningen University, Wageningen, Netherlands

**Keywords:** lactate, glycolysis, cytokines, immunometabolism, innate immune cells, monocytes

## Abstract

Lactate, the end product of anaerobic glycolysis, is produced in high amounts by innate immune cells during inflammatory activation. Although immunomodulating effects of lactate have been reported, evidence from human studies is scarce. Here we show that expression of genes involved in lactate metabolism and transport is modulated in human immune cells during infection and upon inflammatory activation with TLR ligands *in vitro*, indicating an important role for lactate metabolism in inflammation. Extracellular lactate induces metabolic reprogramming in innate immune cells, as evidenced by reduced glycolytic and increased oxidative rates of monocytes immediately after exposure to lactate. A short-term infusion of lactate in humans *in vivo* increased *ex vivo* glucose consumption of PBMCs, but effects on metabolic rates and cytokine production were limited. Interestingly, long-term treatment with lactate *ex vivo*, reflecting pathophysiological conditions in local microenvironments such as tumor or adipose tissue, significantly modulated cytokine production with predominantly anti-inflammatory effects. We found time- and stimuli-dependent effects of extracellular lactate on cytokine production, further emphasizing the complex interplay between metabolism and immune cell function. Together, our findings reveal lactate as a modulator of immune cell metabolism which translates to reduced inflammation and may ultimately function as a negative feedback signal to prevent excessive inflammatory responses.

## Introduction

Intracellular metabolism influences functional properties of immune cells, with high glycolytic rates observed during pro-inflammatory responses (1). Lactate, the end product of the glycolytic route, is produced and secreted in high amounts by innate immune cells upon inflammatory activation (2). Interestingly, high lactate concentrations in tumor microenvironments are known to alter the phenotype of monocytes and macrophages by decreasing cytokine production and migration (3, 4). Apart from its role in the tumor microenvironment, immunomodulatory effects of lactate may also be relevant in the adipose tissue, where concentrations vary dependent on the metabolic state (5) or in the circulation, where lactate levels are known to fluctuate. Circulating lactate levels range from 0.5 to 2 mM and can increase up to 10 to 25 mM after intense exercise (6, 7) or during pathophysiologic conditions, such as sepsis (8), where plasma lactate levels may increase to levels above 4 mM (9, 10).

Various cell types, including innate immune cells, can sense extracellular lactate concentrations via lactate receptors on their cell surface, for instance via the G-protein-coupled receptor GPR81. It has been suggested that GPR81 is required for the inhibitory effects of extracellular lactate on lipopolysaccharide (LPS)-induced IL-1β production of murine macrophages and human PBMCs *in vitro* (11). *In vivo*, anti-inflammatory effects are underscored by studies demonstrating reduced inflammation during hepatitis (11) and prevention of inflammation in a TNBS-induced colitis model (12) after lactate administration. Inhibition of NF-κB and inflammasome activation mediate anti-inflammatory effects of lactate (11), but exact underlying mechanisms remain unknown.

Although various studies have focused on GPR81-mediated effects of lactate, lactate can also be taken up by cells via monocarboxylate transporters (MCTs) and might thereby directly affect cellular metabolism and function. Expression and modulation of MCTs have been shown to be important for immune cell function in T cells and macrophages (13, 14). Lactate treatment decreased glucose uptake of LPS-stimulated human monocytes (15) and high concentrations of extracellular lactate decreased extracellular acidification rates (ECAR) of mouse macrophages independent of GPR81 (16). Together, these results suggest direct effects of lactate on cellular metabolism that might contribute to modulation of immune cell function.

A central enzyme in lactate metabolism is lactate dehydrogenase (LDH). The two different isoforms LDHA and LDHB assemble in five different combinations into tetramers with different kinetic properties. LDHA has a higher affinity for pyruvate compared with lactate, thus converting pyruvate into lactate and NAD^+^, whereas LDHB preferentially converts lactate into pyruvate fueling oxidative metabolism. Whereas LDHA has been implicated in IFNγ-production by T cells (17) and anti-tumor activity of macrophages (18), the function of LDHB in immune cells remains elusive.

We hypothesized that extracellular lactate directly influences intracellular metabolism of human immune cells and thereby also their inflammatory output. We tested this hypothesis by examining the regulation of genes involved in lactate metabolism during immune cell activation. Furthermore, we studied the effects of extracellular lactate on metabolism and function of primary human immune cells isolated from blood of healthy individuals as well as effects of a short-term lactate infusion in people with type 1 diabetes (T1D), a disease associated with inflammatory traits, on *ex vivo* inflammatory responses of immune cells. Our results demonstrate that intracellular lactate metabolism is modulated during inflammatory activation of human immune cells. Furthermore, extracellular lactate affects immune cell metabolism and cytokine production time- and concentration-dependently and may thus serve as a negative feedback signal limiting inflammation.

## Materials and methods

### Healthy volunteers

Buffy coats from healthy donors were obtained after written informed consent (Sanquin Blood Bank, Nijmegen, the Netherlands). Additionally, PBMCs were isolated from fresh blood donated by healthy volunteers. Ethical approval was obtained by the institutional review board of the Radboud university medical center and all participants gave written informed consent before participation.

### Patients with type 1 diabetes

We enrolled twelve patients with type 1 diabetes who participated in a larger study that consisted of either one or two glucose clamp experiments (19). Patients were eligible if they had an HbA_1c_ level < 9.0% (75 mmol/mol) and were free from macrovascular and microvascular complications, except for background retinopathy. Exclusion criteria included a history of cardiopulmonary disease, age >50 years, and the use of drugs other than insulin interfering with glucose metabolism. None of the participants used immunomodulatory drugs. The institutional review board of the Radboud university medical center approved the study and all study participants gave written informed consent before participation.

### *In vivo* lactate infusions

Enrolled patients with type 1 diabetes underwent a stepped hyperinsulinemic-euglycemic-hypoglycemic glucose clamp, as described previously (19). Briefly, insulin was infused continuously (60 mU/m^2^/min) with intravenous administration of glucose 20% at a variable dose, guided by arterial glucose values (Biosen C-line; EKF Diagnostics) measured at 5-min intervals to maintain plasma glucose at 5.0 mmol/l (euglycemia) and 2.8 mmol/l (hypoglycemia), respectively. Approximately 15 min after achieving euglycemia, a primed (40 μmol/kg/min for 15 min) continuous (25 μmol/kg/min) infusion of sodium lactate (600 mmol/L; prepared by the Department of Pharmacy, Radboud university medical center, Nijmegen, The Netherlands) was administered, while plasma glucose levels were maintained at 5 mmol/L. Blood samples for isolation of PBMCs were taken just prior to starting the lactate infusion (when stable euglycemia (blood glucose 5.0 mmol/L) was reached, *T* = 0), and after 25 min of lactate infusion (*T* = 1) and stable euglycemia. For the current study no samples were obtained under hypoglycemic conditions.

### Analytical methods

Peripheral total and differential white blood cell count were determined on a Sysmex XN-450.

### Isolation of PBMCs and CD14^+^ monocytes

Isolation of PBMCs was performed by differential centrifugation over Ficoll-Paque™ PLUS (GE Healthcare Biosciences). CD14^+^ monocytes were purified from freshly isolated PBMCs using MACS microbeads for positive selection, according to the manufacturer's instructions (Miltenyi Biotec). PBMCs from buffycoats were enriched for monocytes by hyperosmotic density gradient centrifugation over Percoll (Sigma-Aldrich) (used for experiments in **Figures 9A–D**).

### Stimulation experiments

For analysis of cytokine release, 5 × 10^5^ PBMCs or 1 × 10^5^ monocytes were used per well in a 96-well plate. Cells were cultured in RPMI 1640 (no glucose, Gibco) supplemented with 50 μg/mL gentamycin (Gibco), 1 mM pyruvate (Gibco), 10 mM HEPES (Sigma-Aldrich), 5.5 mM glucose (Sigma-Aldrich). If indicated cells were pretreated with sodium lactate (used for *in vivo* experiments and described above) and stimulated with either medium, 10 ng/mL of the TLR4 agonist lipopolysaccharide (LPS) from *Escherichia coli* (Sigma-Aldrich) or 10 μg/mL of the TLR2 agonist Pam_3_CysSK_4_ (Pam3Cys) (EMC Microcollections). If indicated α-cyano-4-hydroxycinnamic acid (Sigma-Aldrich) or sodium oxamate (Sigma-Aldrich) were added prior to the stimulation.

### Cytokine measurements

The production of interleukin (IL)-1β, IL-6, tumor necrosis factor (TNF)-α (R&D Systems), and IL-10 (Sanquin) was measured by ELISA.

### Glucose measurements

Glucose concentrations were measured in cell culture supernatants. Measurements were based on an enzymatic reaction in which glucose is oxidized and the resulting H_2_O_2_ is coupled to the conversion of Amplex Red reagent to fluorescent resorufin by horseradish peroxidase. The fluorescence of resorufin (excitation/emission maxima 570/585 nm) was measured on a 96-well plate reader (BioTek).

### Extracellular flux analysis

Real-time OCR and ECAR of monocytes were analyzed using a XF-96 Extracellular Flux Analyzer (Seahorse Bioscience). Basal metabolic rates of monocytes seeded in quintuplicate were determined during consecutive measurements in unbuffered Seahorse medium (8.3 g DMEM powder, 0.016 g phenol red and 1.85 g NaCl in 1 L water, pH set at 7.4 at 37°C; sterile filtered) containing 5.5 mM glucose and 2 mM L-glutamine. After three basal measurements, subsequent measurements were performed following the addition of medium containing either 0, 3.5, or 15 mM Na-L-lactate. If indicated, mitochondrial metabolism was assessed by the subsequent injection of oligomycin A (1 μM), FCCP (1 μM) together with pyruvate (1 mM) and antimycin A (2.5μM) together with rotenone (1.25μM) (all from Sigma-Aldrich). Flux measurements were normalized to total DNA content using a Quant-iT dsDNA high sensitivity assay kit (Thermofisher Scientific). The 96-well plates for Seahorse measurements were pretreated with Cell-Tak Cell and Tissue Adhesive (Corning).

### RNA isolation and RT-PCR

For mRNA expression analysis, cells were lysed in TRIzol reagent (Invitrogen) and RNA isolation was performed according to the manufacturer's instructions. RNA was transcribed into complementary DNA by reverse transcription using the iScript cDNA synthesis kit (Bio-Rad). Power SYBR Green PCR Master Mix (Applied Biosystems) was used for semiquantitative RT-PCR in a StepOnePlus™ Real-Time PCR System (Applied Biosystems). Expression data were normalized to the housekeeping gene β_2_*M*. Primers with the following sequences were used: B2M_forward ATGAGTATGCCTGCCGTGTG, B2M_reverse CCAAATGCGGCATCTTCAAAC, LDHA_forward ATGGCAACTCTAAAGGATCAGC, LDHA_reverse CCAACCCCAACAACTGTAATCT, LDHB_forward TCCGCACGACTGTTACAGAG, LDHB_reverse TTGCCTCTTCTTCCGCAACT.

### Metabolite measurements

**Figure 1:**
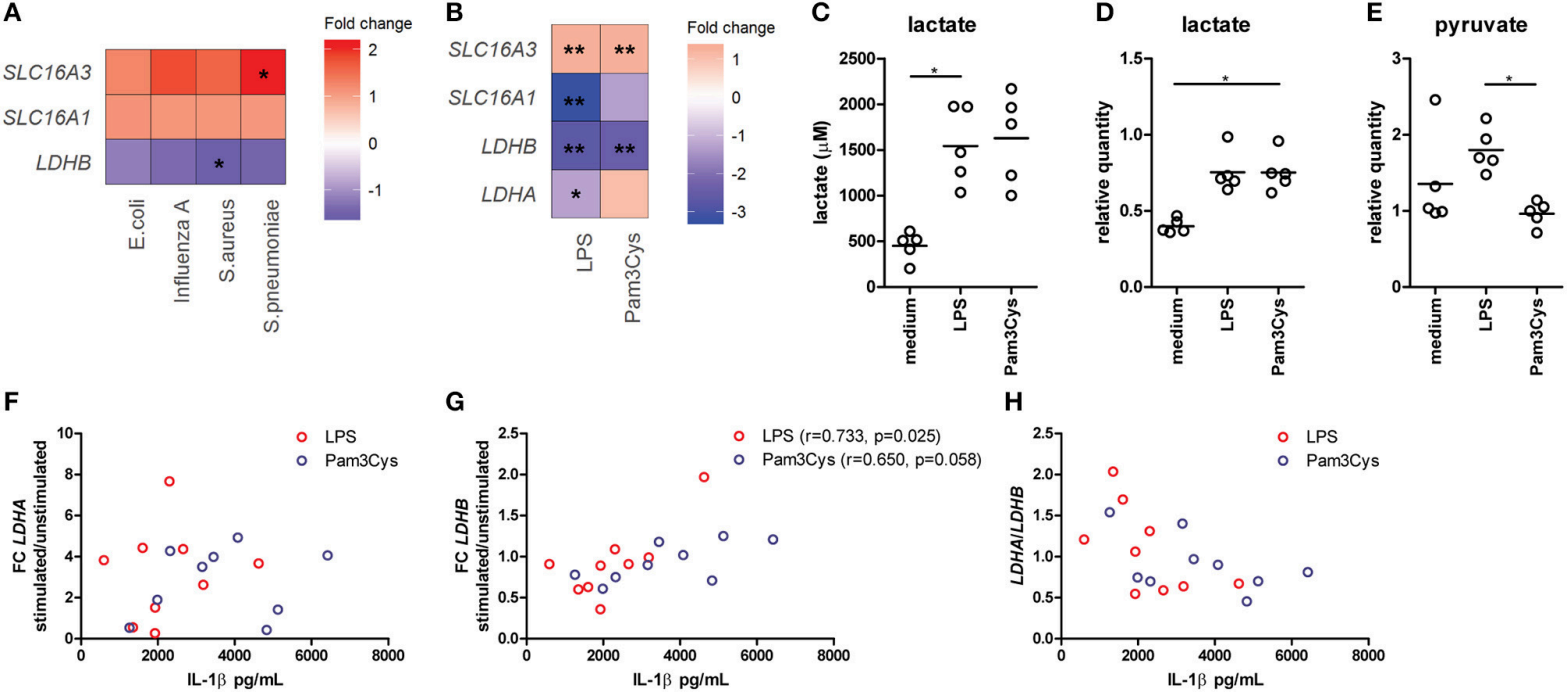
Lactate dehydrogenase and lactate transporters are important for inflammatory responses of human immune cells. **(A)** Gene expression analysis from microarray data of PBMCs isolated from patients with acute infections. Fold change of gene expression from PBMCs isolated from patients with infections compared with healthy controls is shown. **(B)** Gene expression analysis from microarray data of human monocytes stimulated for 24 h with LPS or Pam3Cys. Fold change of gene expression from monocytes stimulated with LPS or Pam3Cys compared with unstimulated cells is shown. **(C)** Lactate levels measured in supernatants of CD14^+^ monocytes stimulated for 24 h with LPS or Pam3Cys. **(D,E)** Relative quantity of intracellular lactate **(D)** and pyruvate **(E)** in CD14^+^ monocytes stimulated for 24 h with LPS or Pam3Cys. **(F–H)** Correlation of the change in LDH expression upon LPS- or Pam3Cys-stimulation with IL-1β production. *p < 0.05, **p < 0.01. Friedman test with *post-hoc* Dunn's test **(C–E)**. Spearman's rank test **(F–H)**.

Metabolite quantities displayed in Figures 1D,E were retrieved from metabolome analysis of CD14^+^ monocytes stimulated with LPS or Pam3Cys as described earlier (20). Data from metabolite measurements normalized to protein concentration was used.

### Microarray analysis

Expression changes in PBMCs isolated from patients (mostly pediatric) with acute infections (confirmed microbiologic diagnosis of *Escherichia coli, Staphylococcus aureus, Streptococcus pneumonia* or influenza A) were retrieved from publically available microarray data (GSE6269) (21). Expression changes in human monocytes stimulated with LPS and Pam3Cys were retrieved from (GSE78699) (20). Arrays were normalized using the robust multiarray average method (22, 23). Probe sets were defined according to the method of Dai et al. (24). In this method, probes are assigned to Entrez IDs as a unique gene identifier. The *p*-values were calculated using an intensity-based moderated t-statistic (25).

### Statistical analysis

Differences between two variables were compared with the paired Wilcoxon's signed rank test. For comparison of 3 or more variables Friedman's test with *post-hoc* Dunn's test (for selected pairs) was used. For correlation analysis Spearman's rank test was used. All data are expressed as mean ± standard deviation (SD), unless otherwise specified. A *p*-value < 0.05 was considered statistically significant. Statistical analyses were performed with *GraphPad Prism* or *IBM SPSS Statistics 22*. Heatmaps were generated in *R studio* using *ggplot2* or the *corrplot* package.

## Results

### Lactate metabolism is involved in inflammatory responses of human immune cells

Gene expression of the lactate transporter MCT4 (*SLC16A3*) is upregulated, whereas *LDHB* expression is downregulated in PBMCs isolated from patients with acute infections compared with healthy controls (Figure 1A), indicating an important role for lactate metabolism in circulating immune cells during inflammation. Expression of the lactate transporter MCT1 (*SLC16A1*) was not affected significantly. To further decipher the potential role of lactate metabolism for human immune cells, we analyzed gene expression in monocytes stimulated with TLR-ligands *in vitro*. Stimulation of human monocytes with the TLR4-agonist LPS and the TLR2-agonist Pam3CysSK_4_ (Pam3Cys) both increased gene expression of *SLC16A3* and decreased expression of *LDHB* compared with unstimulated cells (Figure 1B). *LDHA* and *MCT1* expression were decreased in monocytes stimulated with LPS, but not affected significantly in cells stimulated with Pam3Cys. In line with these findings, we found increased extra- and intracellular lactate levels in monocytes stimulated with LPS or Pam3Cys compared with unstimulated cells (Figures 1C,D). Interestingly, intracellular pyruvate levels were higher in LPS- compared with Pam3Cys-stimulated monocytes (Figure 1E). Different pyruvate/lactate ratios may indicate differential activation of LDH in LPS- vs. Pam3Cys-stimulated cells. The change in *LDHB*, but not *LDHA*, expression upon stimulation of PBMCs correlated significantly with IL-1β production (Figures 1F–H), indicating an interaction of lactate metabolism, especially *LDHB*, and immune cell function.

### Lactate acutely affects metabolism of human monocytes *in vitro*

**Figure 2:**
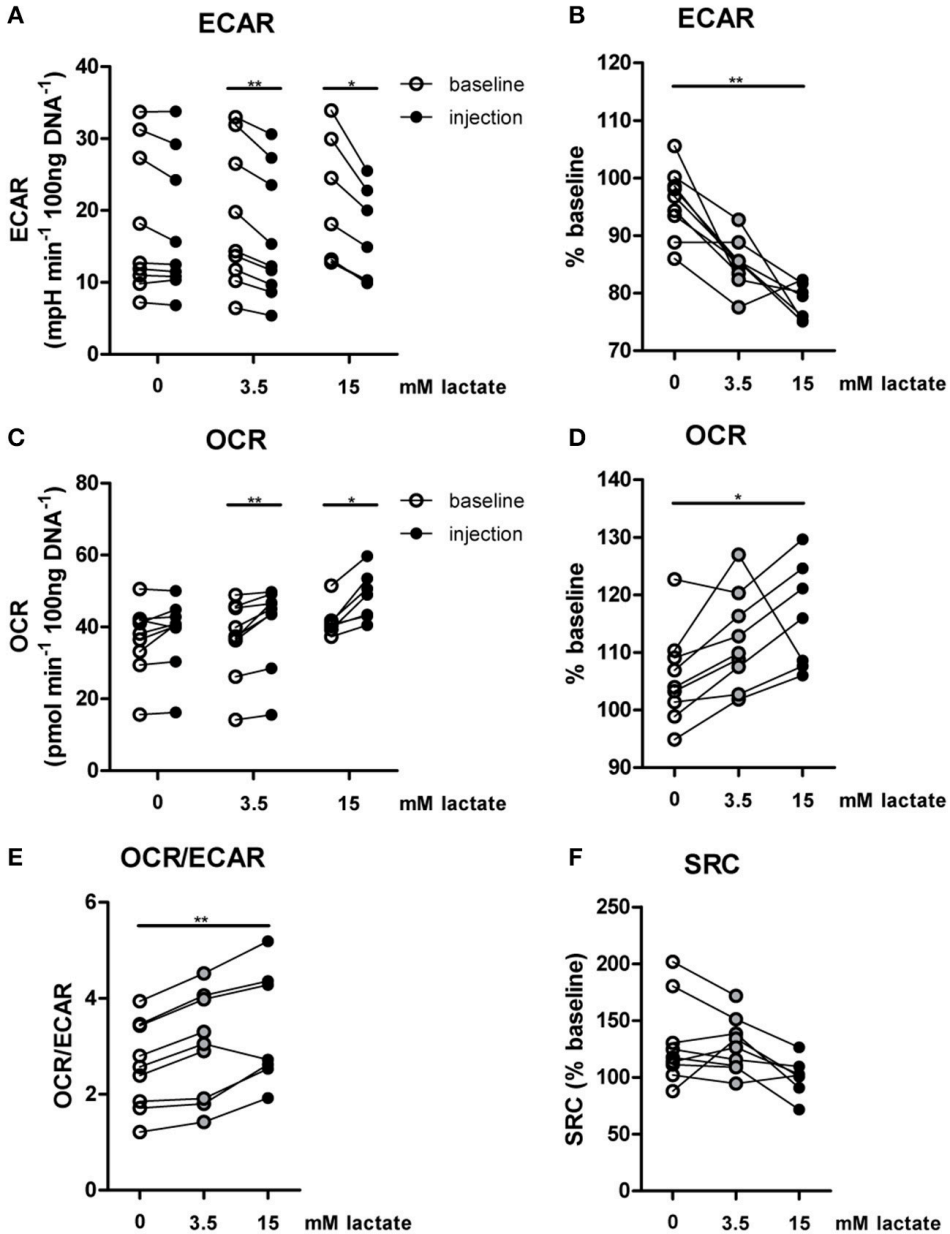
*In vitro*, lactate acutely shifts metabolism of human monocytes to oxidative phosphorylation. **(A–D)** Change of ECAR **(A,B)** and OCR **(C,D)** in human CD14^+^ monocytes after exposure to lactate, presented as raw data or % baseline (before lactate injection). **(E)** OCR/ECAR after lactate injection. **(F)** Spare respiratory capacity (SRC) after lactate injection. Each dot represents one healthy individual. *n* = 7–9, *p < 0.05, **p < 0.01, Wilcoxon signed rank test **(A,C)**, Friedman test with *post-hoc* Dunn's test **(B,D–F)**.

**Figure 3:**
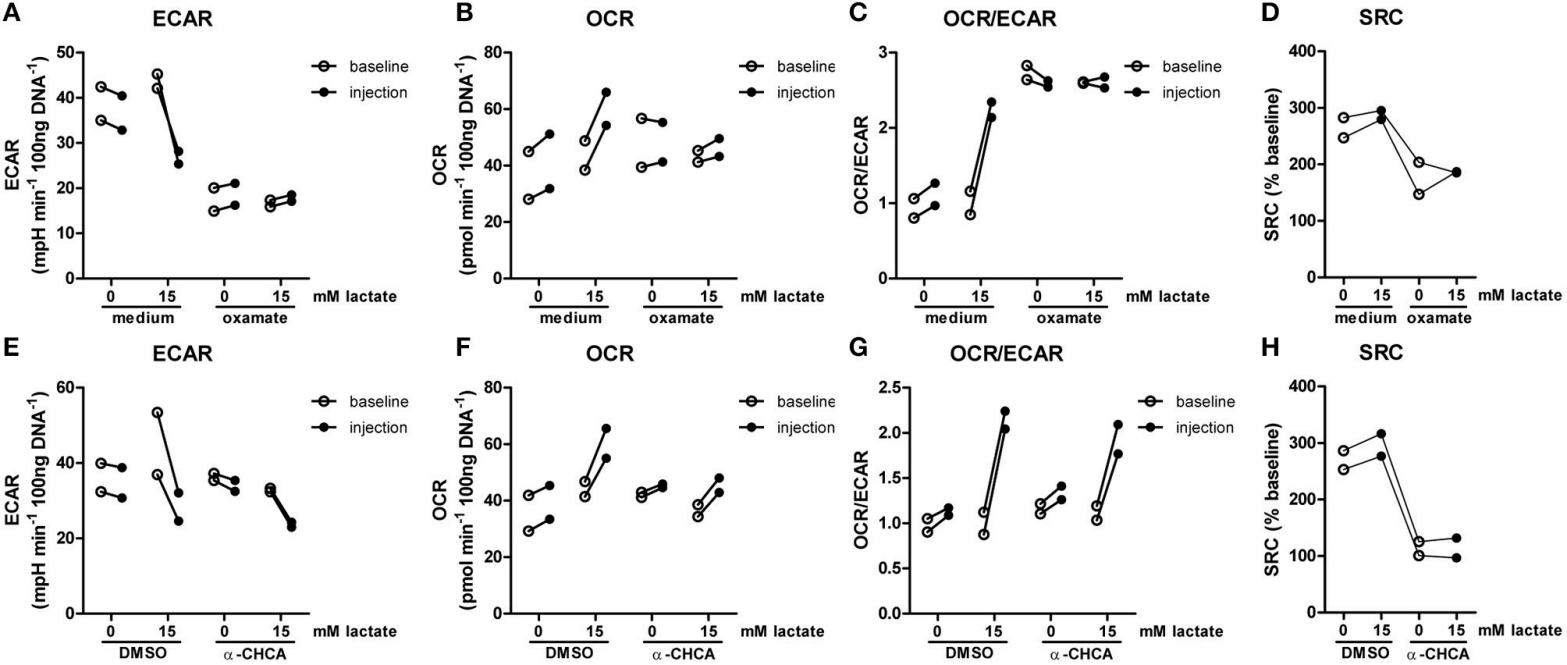
LDH-inhibition abolishes acute effects of lactate on metabolism. CD14^+^ monocytes were pretreated for 1 h with 40 mM sodium oxamate and medium as a control **(A–D)** or with 0.5 mM α-cyano-4-hydroxycinnamic acid (α-CHCA) and DMSO as a control **(E–H)** before cells were exposed to lactate and metabolism was assessed. Change of ECAR **(A,E)** OCR **(B,F)** OCR/ECAR **(C,G)** and SRC **(D,H)** in human CD14^+^ monocytes after exposure to lactate. Each dot represents one healthy individual. *n* = 2.

The importance of LDH and MCTs in regulating inflammatory responses is indicative for a role of lactate in modulating immune cell metabolism. Recently, it was demonstrated that very high concentrations of lactate (68 mM) immediately inhibit glycolysis of murine macrophages (16), but whether this effect is relevant for human immune cells at physiologic concentrations remains to be determined. Therefore, we investigated direct effects of Na^+^-lactate at concentrations occurring in the circulation after exercise or in pathophysiologic conditions (3.5 and 15 mM) on cellular metabolism of CD14^+^ monocytes isolated from blood of healthy volunteers. Lactate concentrations in this range decreased cytokine production by murine macrophages *in vitro* and plasma lactate levels of 3 mM suppressed inflammation in mice *in vivo* (11). Exposure to lactate immediately reduced extracellular acidification rate (ECAR) (Figures 2A,B) and increased oxygen consumption rate (OCR) (Figures 2C,D) of monocytes dose-dependently. Overall, this resulted in an acutely increased OCR/ECAR ratio after exposure of cells to lactate (Figure 2E), a metabolic state characteristic for anti-inflammatory responses. Lactate did not induce acute changes in spare respiratory capacity (SRC), implying no acute changes in mitochondrial capacity to respond to a sudden increase in energy demand (Figure 2F). Acute effects of extracellular lactate on monocyte metabolism were abolished, if cells were pretreated with the LDH-inhibitor oxamate (Figures 3A–D). The MCT-inhibitor α-cyano-4-hydroxycinnamic acid (α-CHCA) however only had limited effects on metabolic changes induced by lactate (Figures 3E–H).

**Figure 4:**
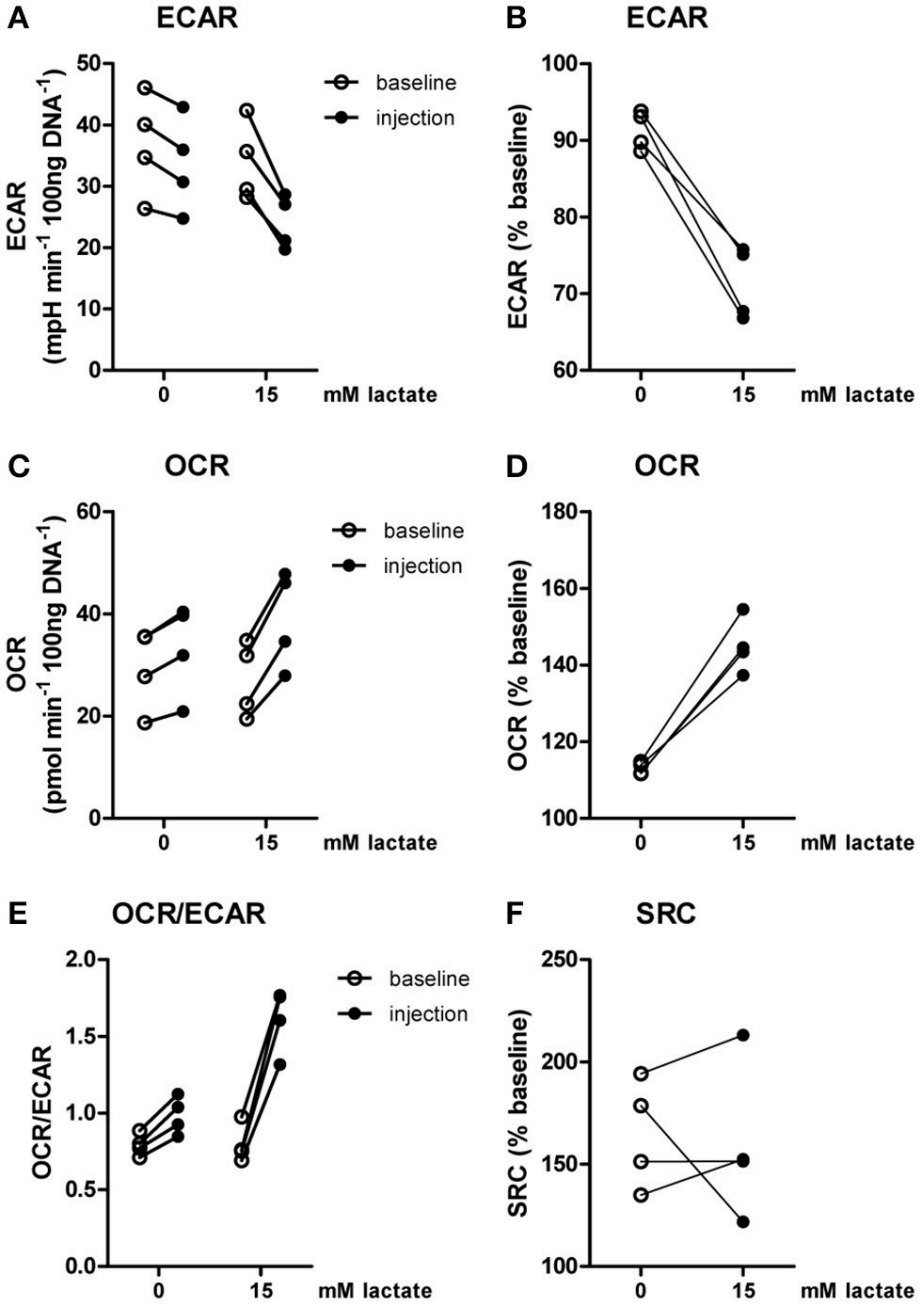
Lactate acutely shifts metabolism of inflammatory human monocytes to oxidative phosphorylation. CD14^+^ monocytes were stimulated with LPS for 4 h before metabolism was assessed. **(A–D)** Change of ECAR **(A,B)** and OCR **(C,D)** in human CD14^+^ monocytes stimulated for 4 h with LPS before exposure to lactate, presented as raw data or % baseline (before lactate injection). **(E)** OCR/ECAR after lactate injection. **(F)** Spare respiratory capacity (SRC) after lactate injection. Each dot represents one healthy individual. *n* = 4, Wilcoxon signed rank test.

Inflammatory conditions alter lactate metabolism and may thus affect metabolic reprogramming induced by extracellular lactate. When lactate was added under inflammatory conditions (4 h after stimulation with LPS), ECAR was decreased (Figures 4A,B) and OCR (Figures 4C,D) was increased, resulting in an increased OCR/ECAR ratio (Figure 4E). SRC was not affected by acute exposure to lactate (Figure 4F).

### A short-term lactate infusion mildly affects glycolytic metabolism of human PBMCs *ex vivo*

**Figure 5:**
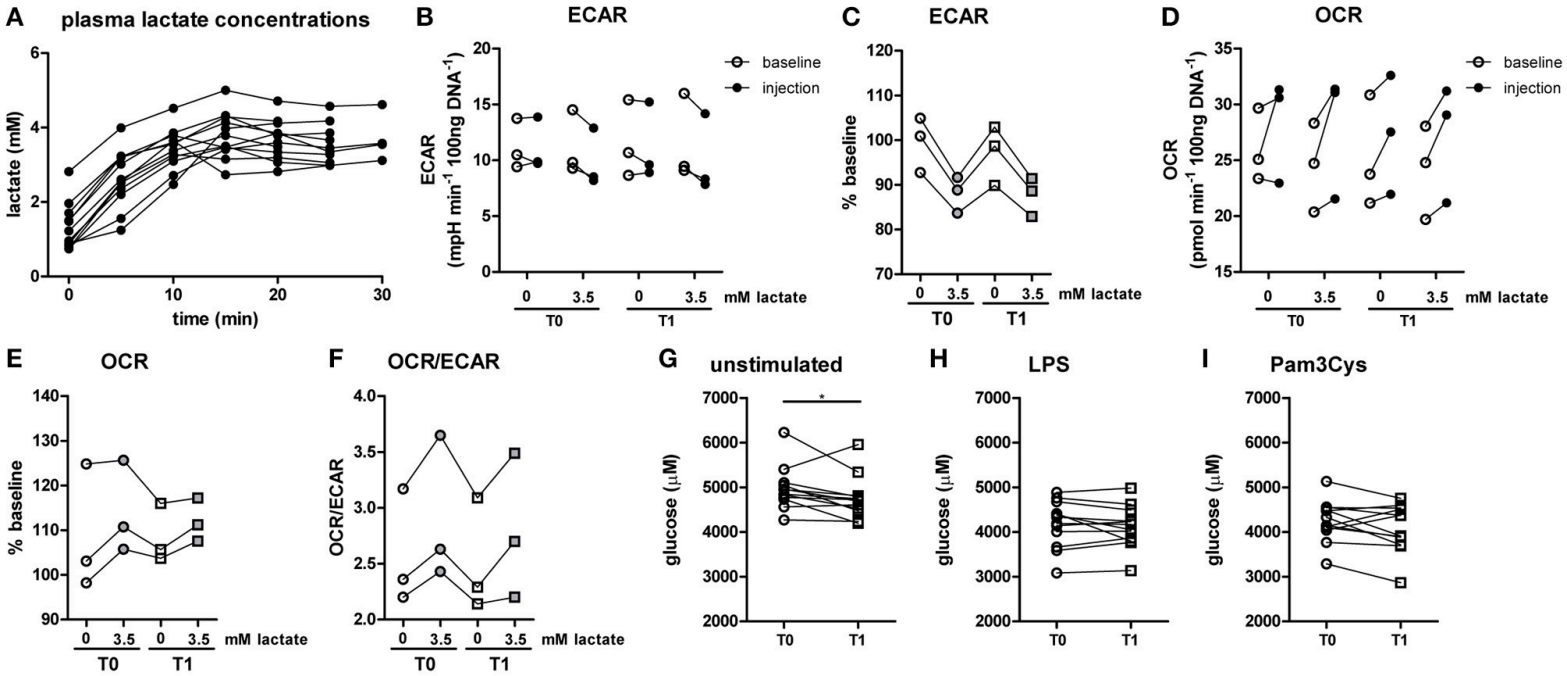
A short-term lactate infusion only has minor effects on human immune cell metabolism. **(A)** Plasma lactate concentrations measured during lactate infusion. **(B–E)** Change of ECAR **(B,C)** and OCR **(D,E)** in human CD14^+^ monocytes after exposure to lactate, presented as raw data and % baseline (before lactate injection). **(F)** OCR/ECAR after lactate injection. **(G–I)** Glucose concentration assessed in the medium of cultured PBMCs from patients with diabetes. PBMCs were isolated and stimulated either before (T0) or after (T1) lactate infusion. PBMCs were stimulated with LPS or Pam3Cys for 24 h. Each dot represents one patient. **(A,G–I)** *n* = 12, **(B–F)** *n* = 3. *p < 0.05, Friedman test with *post-hoc* Dunn's test.

Since lactate immediately affected human immune cell metabolism *in vitro*, we investigated whether a short-term lactate infusion *in vivo* also affects immune cell metabolism and possibly function. Due to the potential anti-inflammatory effects of lactate, the infusion was performed in patients with type 1 diabetes (T1D) (see Table 1 for patient characteristics), a condition frequently accompanied by the presence of a low grade inflammatory state (26). Consequently, we expected pronounced anti-inflammatory effects of lactate in patients with T1D. Lactate was infused during stable euglycemia (5.1 ± 0.2 mmol/L) and increased plasma lactate levels from 1.3 ± 0.6 mM at baseline to 3.7 ± 0.5 mM after 20 min of infusion (*p* = 0.002, Figure 5A).

**Table 1:** Baseline characteristics in people with diabetes.

	**Total (*n* = 12)**	**Male (*n* = 6)**	**Female (*n* = 6)**
Age (years)	26.3 ± 7.8	29.3 ± 9.6	23.3 ± 4.3
BMI (kg/m^2^)	23.9 ± 2.2	24.5 ± 1.8	23.3 ± 2.6
Duration of diabetes (years)	11.3 ± 4.4	8.2 ± 3.4	14.5 ± 2.8
**HbA1c**
%	7.1 ± 0.6	6.9 ± 0.5	7.3 ± 0.8
mmol/mol	53.8 ± 7.1	51.5 ± 5.4	56.2 ± 8.2

*Data are means ± SD*.

In line with results in healthy individuals (Figure 2), we found that lactate treatment *in vitro* acutely increased the OCR/ECAR ratio of monocytes from patients with T1D (Figures 5B–F). We did not find significant differences in metabolism between cells isolated before (T0) and directly after lactate infusion (T1). However, lactate infusion increased glucose consumption of unstimulated cells (Figure 5G). Interestingly, glucose consumption of cells stimulated with LPS or Pam3Cys were not affected (Figures 5H,I).

### A short-term lactate infusion only mildly affects *ex vivo* cytokine production of PBMCs

**Figure 6:**
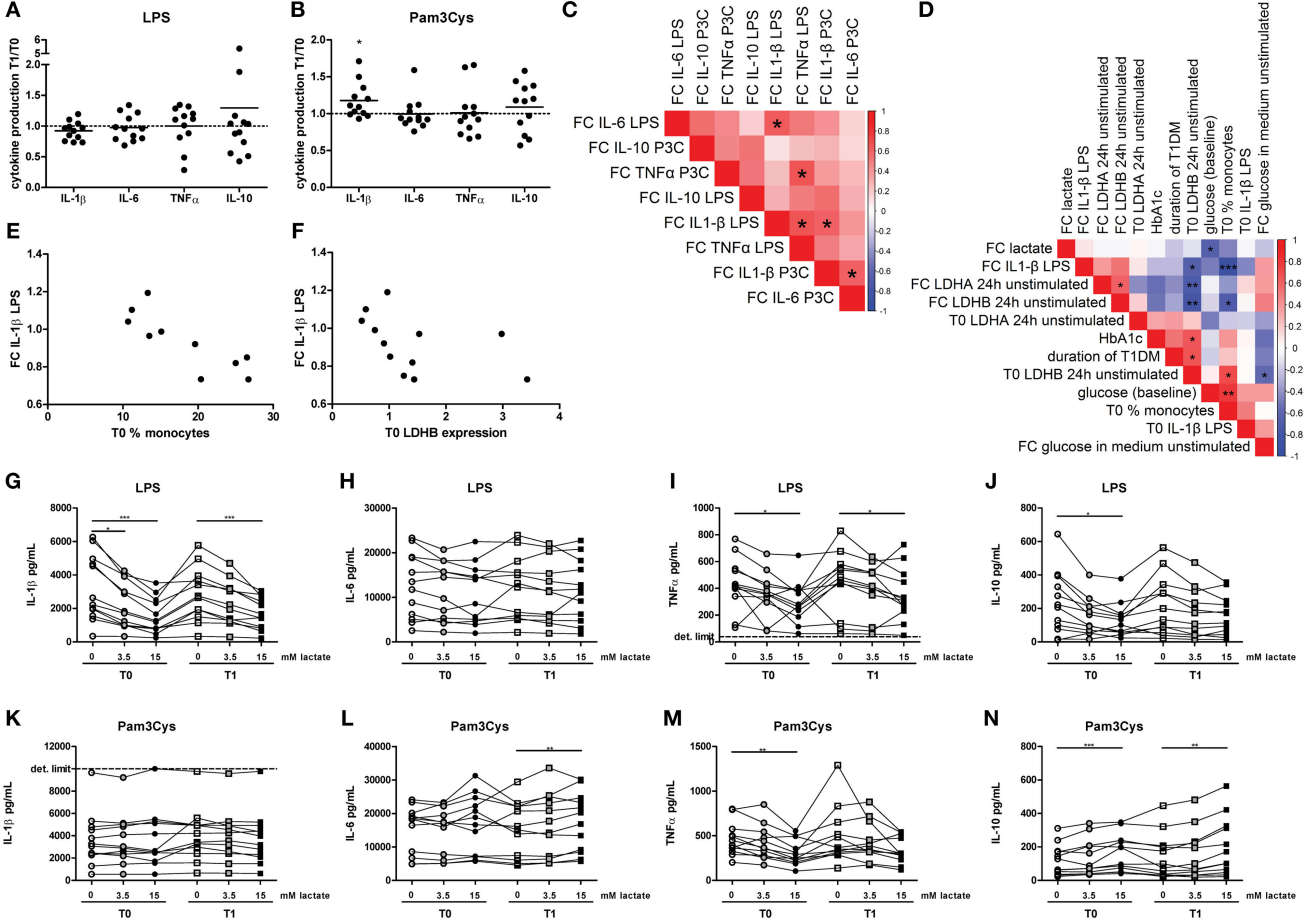
A short-term lactate infusion only has minor effects on cytokine production of human PBMCs. PBMCs were isolated and stimulated either before (T0) or after (T1) lactate infusion. **(A,B)** Change in LPS- **(A)** and Pam3Cys- **(B)** induced cytokine production after lactate infusion. **(C)** Correlation heatmap indicating correlations between changes in cytokine production after lactate infusion. Colors indicate Spearman correlation coefficients. FC, Fold change T1/T0. **(D)** Correlation heatmap indicating correlations between changes in LPS-induced IL-1β production after lactate infusion and various clinical and experimental factors. **(E,F)** Correlation of changes in LPS-induced IL-1β production after lactate infusion with percentage of monocytes in the PBMC fraction **(E)** and gene expression levels of LDHB **(F)**. **(G–N)** Cytokine production of human PBMCs pretreated with lactate for 1 h before LPS **(G–J)** or Pam3Cys **(K–N)** was added for 24 h. *n* = 12. *p < 0.05, **p < 0.01, ***p < 0.001. Wilcoxon signed rank test **(A,B)** Spearman's rank test **(C–F)** Friedman test with *post-hoc* Dunn's test **(G–N)**.

Although effects on glycolytic metabolism were minor, we tested whether lactate infusion modulated inflammatory responses of immune cells. In line with mild effects on metabolism, the short-term lactate infusion did not affect LPS-induced cytokine production *ex vivo* significantly (Figure 6A). Since lactate may affect immune cell metabolism, and metabolic pathways are differentially regulated by different TLR stimulations (20), PBMCs were also stimulated with Pam3Cys. Surprisingly, *in vivo* administration of lactate even mildly increased Pam3Cys-induced IL-1β production (Figure 6B).

We observed substantial variation in the effect of lactate infusion on *ex vivo* cytokine production between different donors. To determine if the effect of lactate infusion on different cytokines was similar within one individual, we analyzed correlations between changes in different cytokines. Changes in LPS-induced IL-1β production after lactate infusion correlated with changes in LPS-induced IL-6 and TNF-α as well as Pam3Cys-induced IL-1β production (Figure 6C), indicating that lactate infusion had consistent effects on different cytokines within one person.

Differential effects of the lactate infusion in different individuals may be due to different plasma lactate concentrations during the infusion, but might also be influenced by differences in glucose control and diabetes duration or differences in the inflammatory status at baseline. There was no difference in the effect of the lactate infusion between male and female participants (data not shown). We did not find any correlation of baseline lactate concentrations or the change in plasma lactate concentrations with the changes of the inflammatory response upon infusion (Figure 6D). Effects of the lactate infusion also did not depend on clinical parameters including glucose levels, HbA1c or duration of diabetes. However, the reduction in LPS-induced IL-1β production after lactate infusion correlated with the number of monocytes within the PBMCs before lactate infusion (Figures 6D,E) and with unstimulated gene expression levels of LDHB (Figures 6D,F), indicating a role for lactate metabolism in conveying the anti-inflammatory effects of lactate.

Anti-inflammatory effects of lactate may be concentration-dependent as already suggested by acute effects of lactate on cellular metabolism (Figure 2), and may as well be dependent on the duration of exposure to lactate. Long-term exposure to lactate is particularly relevant in microenvironments with high local lactate concentrations, for example in the tumor microenvironment or in the adipose tissue. Addition of lactate to the culture medium for 24 h did not induce any cytokine production above the detection limit in unstimulated cells (data not shown), but reduced LPS-induced IL-1β, TNFα, and IL-10 production dose-dependently (Figures 6G–J). In contrast to LPS-induced cytokine production, lactate reduced Pam3Cys-induced TNFα production, but did not affect IL-1β production and even increased IL-10 production (Figures 6K–N). Similar effects were seen in PBMCs isolated after *in vivo* lactate infusion.

### Lactate decreases pro-inflammatory cytokine production of human PBMCs *in vitro*

**Figure 7:**
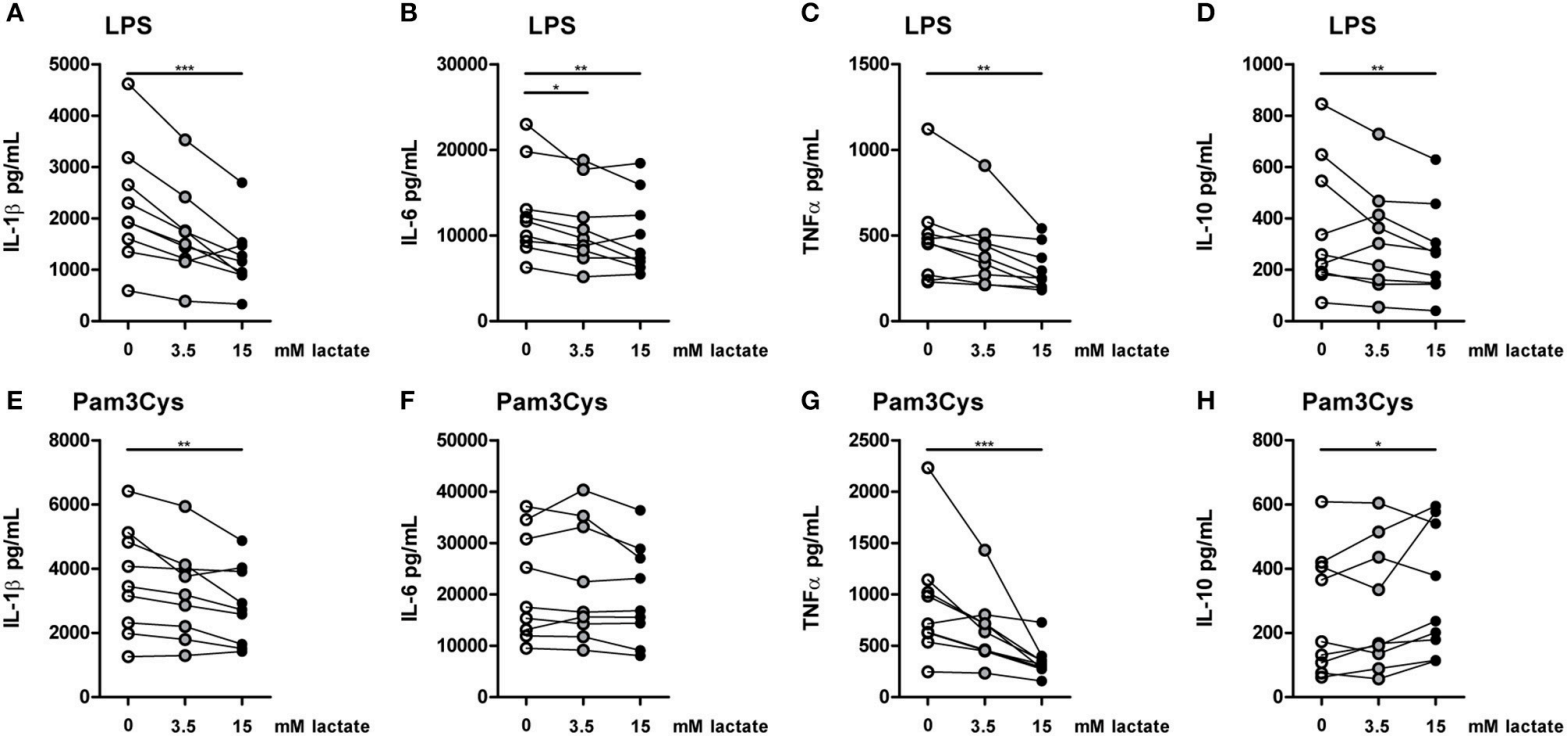
*In vitro*, lactate has anti-inflammatory effects on PBMCs. Cytokine production of PBMCs from healthy individuals (4 men, 5 women). PBMCs were pretreated with lactate for 1 h before LPS **(A–D)** or Pam3Cys **(E–H)** was added for 24 h. **(A–H)** *n* = 9. *p < 0.05, **p < 0.01, ***p < 0.001, Friedman test with *post-hoc* Dunn's test.

**Figure 8:**
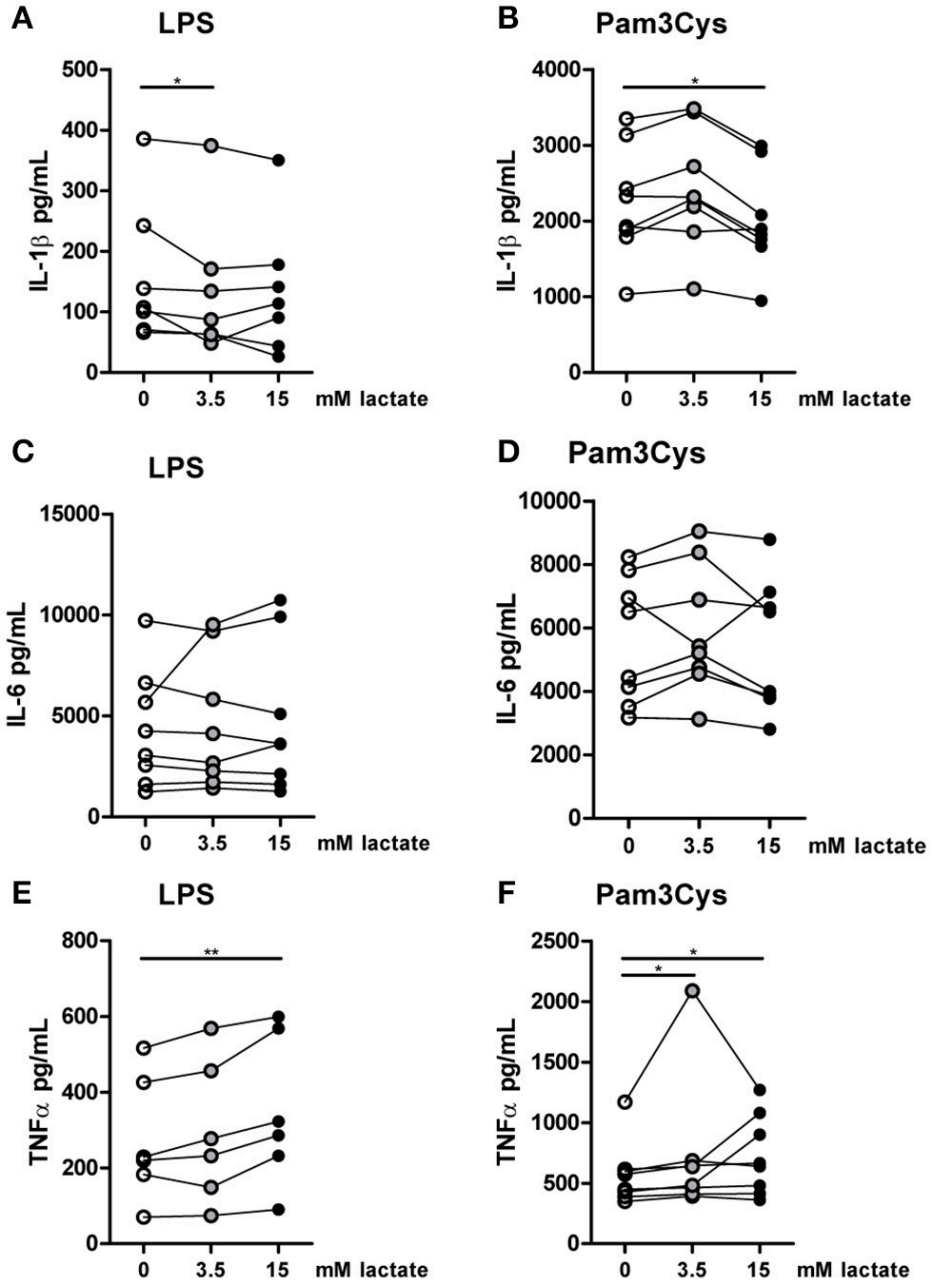
*In vitro*, lactate reduces IL-1β production of monocytes. Cytokine production of CD14^+^ monocytes from healthy individuals. Monocytes were pretreated with lactate for 1 h before LPS **(A,C,E)** or Pam3Cys **(B,D,F)** was added for 24 h. **(A–F)** *n* = 6–8. *p < 0.05, **p < 0.01, Friedman test with *post-hoc* Dunn's test.

To further explore effects of long-term exposure to lactate, we investigated whether extracellular lactate has similar effects on *ex vivo* stimulations of PBMCs isolated from healthy individuals compared with T1D patients. Addition of lactate reduced LPS-induced IL-1β, IL-6, TNFα, and IL-10 production (Figures 7A–D). When stimulated with Pam3Cys, immunomodulatory effects of lactate were less pronounced. Although lactate decreased Pam3Cys-induced IL-1β and TNFα production (Figures 7E,G), it did not affect IL-6 (Figure 7F) and even increased IL-10 production (Figure 7H), suggesting stimulus-dependent effects of lactate metabolism. Overall, *in vitro* effects of lactate on cytokine production were comparable between patients with T1D and healthy individuals and differed mainly in the effect of lactate on Pam3Cys-induced IL-1β production. PBMCs consist of several cell types, but the measured cytokines are likely monocyte-derived. This is also suggested by a strong correlation between cytokine production by PBMCs and the percentage of monocytes within PBMCs (in patients: *r* = 0.72 and *p* = 0.02 for LPS-stimulated IL-6; *r* = 0.79 and *p* = 0.009 for Pam3Cys-induced IL-1β; *r* = 0.65 and *p* = 0.05 for Pam3Cys-induced IL-6). Similar to effects in PBMCs, we also observed decreased IL-1β production in monocytes upon exposure to lactate (Figures 8A,B). Extracellular lactate did not affect IL-6 (Figures 8C,D) production of monocytes and even increased production of TNFα (Figures 8E,F).

### Immunomodulating effects of lactate are time-dependent

**Figure 9:**
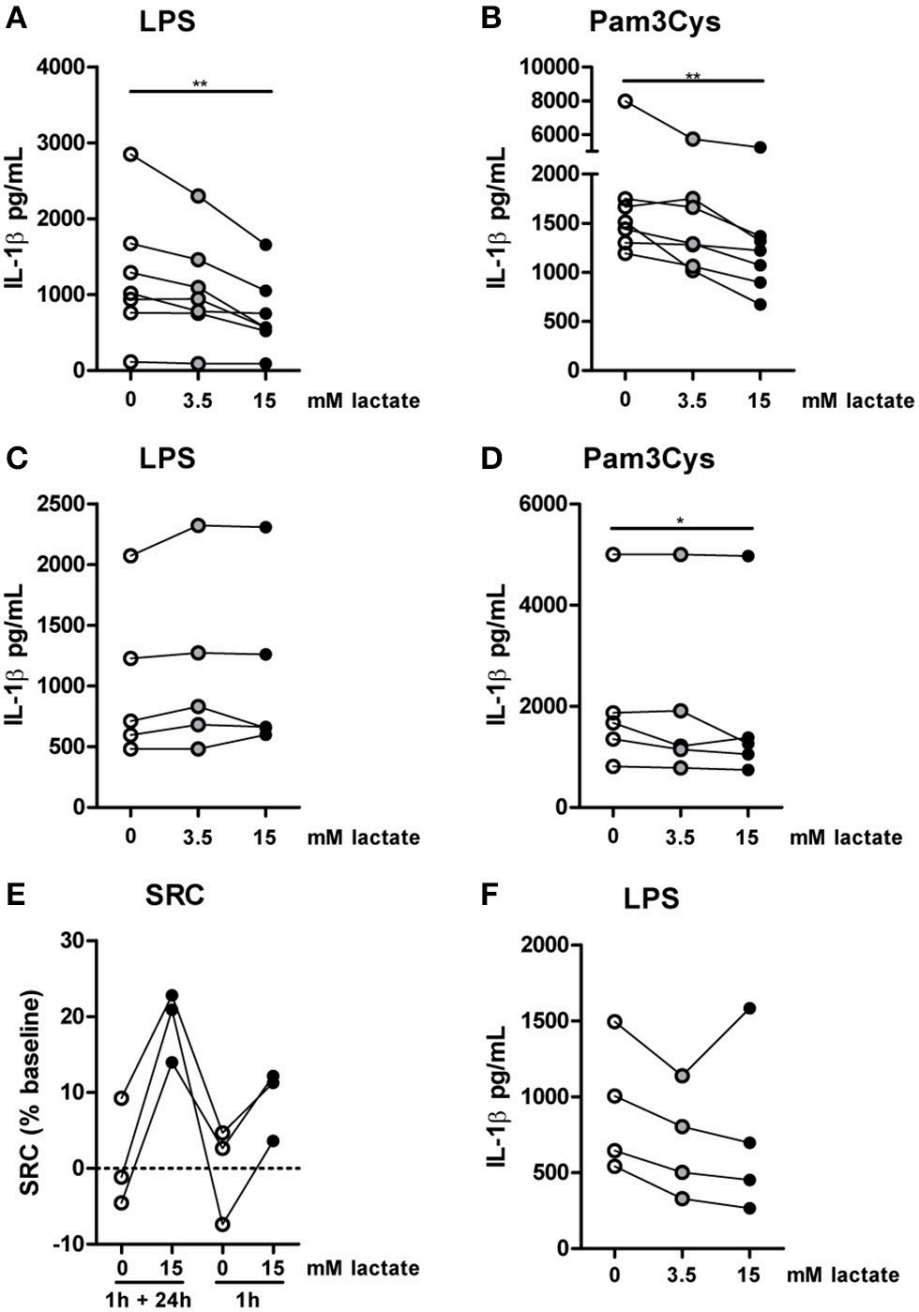
Anti-inflammatory effects of lactate are time-dependent. Cytokine production of monocytes from healthy individuals. **(A,B)** Monocytes were pretreated with lactate for 1 h before LPS **(A)** or Pam3Cys **(B)** was added for 24 h. **(C,D)** Monocytes were pretreated with lactate for 1 h, lactate was removed and cells were washed once before LPS **(C)** or Pam3Cys **(D)** was added for 24 h. **(E)** Monocytes were treated as described for **(A)** or **(C)** before metabolism was measured. **(F)** Monocytes were stimulated with LPS. 4 h after stimulation, lactate was added. **(A,B)** *n* = 7, **(C,D)** *n* = 5, **(E)** *n* = 3, **(F)** *n* = 4. *p < 0.05, **p < 0.01, Friedman test with *post-hoc* Dunn's test.

Compared with the strong effects of lactate on metabolism and cytokine production *in vitro*, changes in *ex vivo* metabolism and cytokine production after lactate infusion were minor. Since lactate exposure induces rapid changes in metabolism, removing the potential substrate may lead to rapid reversal of the induced changes, reflecting metabolic flexibility of human immune cells depending on nutrient availability. We therefore tested, whether lactate also affected cytokine production, when it was removed before starting the stimulation. Long-term exposure to lactate reduced IL-1β production of monocytes (Figures 9A,B), but when monocytes were exposed to lactate for 1h, and lactate was washed away before stimulation with LPS or Pam3Cys, anti-inflammatory effects of lactate were abolished (Figures 9C,D). This suggests restored immune cell function after removal of lactate. Limited effects of short-term lactate treatment on monocyte function are also reflected in metabolism. LPS-stimulated monocytes rely mainly on glycolytic metabolism and have low oxidative capacity reflected in low SRC. Whereas long-term exposure to lactate during LPS treatment increased SRC, a short-term exposure to lactate only had mild effects on SRC of LPS-stimulated cells (Figure 9E). The observation that lactate could still affect metabolism (Figure 4) and reduced cytokine production when added 4h after LPS (Figure 9F), suggests that the presence of lactate during the stimulation may be crucial for its anti-inflammatory effects.

### Inhibition of lactate metabolism modulates cytokine production

**Figure 10:**
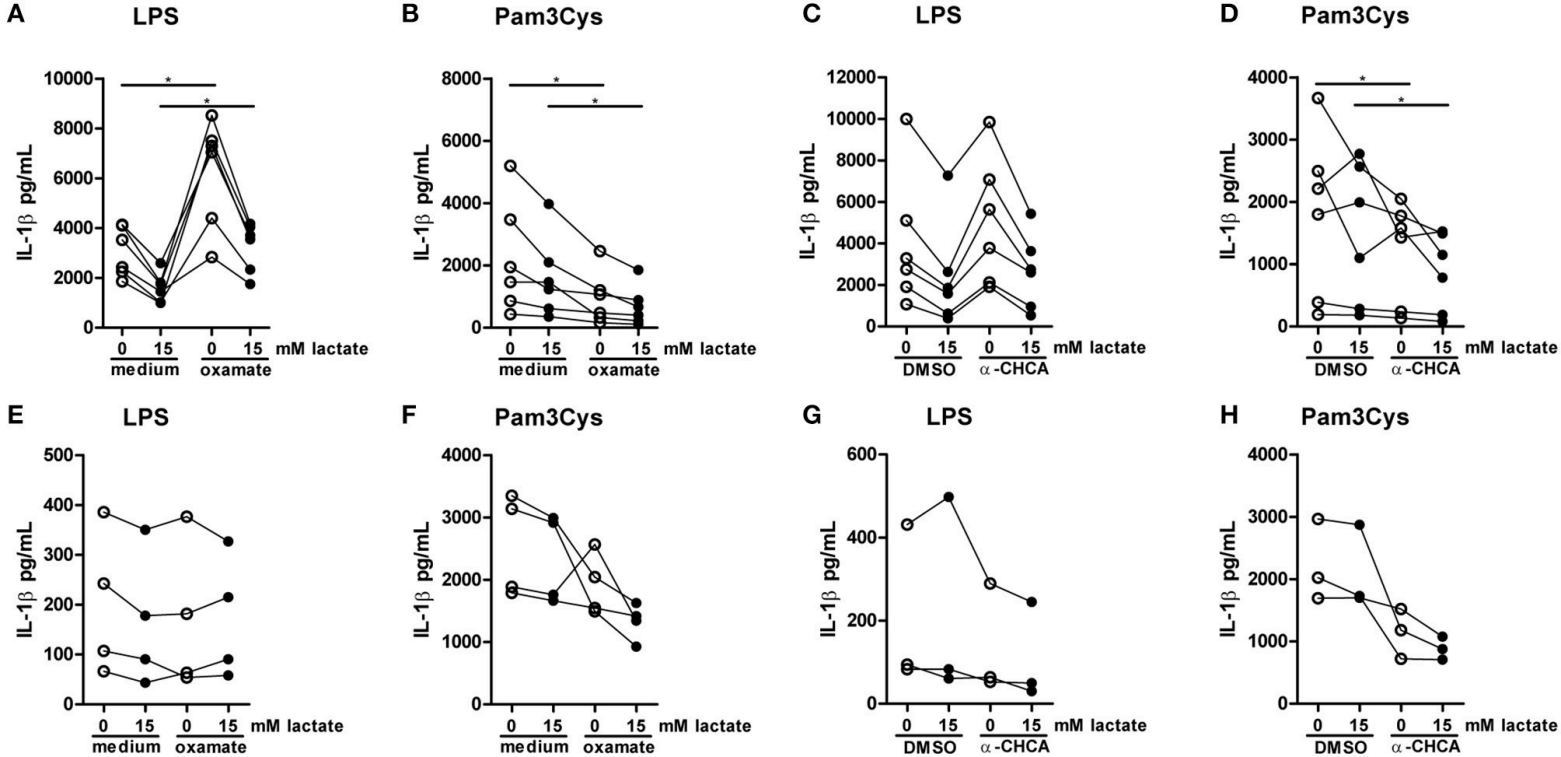
Inhibition of lactate metabolism modulates cytokine production. **(A–D)** Cytokine production of PBMCs from healthy individuals. PBMCs were pretreated with 40 mM sodium oxamate and medium as a control **(A,B)** or 0.5 mM α-cyano-4-hydroxycinnamic acid (α-CHCA) and DMSO as a control **(C,D)** for 1 h, before first lactate was added for 1 h and then LPS **(A,C)** or Pam3Cys **(B,D)** was added for 24 h. **(E–H)** Cytokine production of CD14^+^ monocytes from healthy individuals. Monocytes were pretreated with 40 mM sodium oxamate and medium as a control **(E–F)** or 0.5 mM α-CHCA and DMSO as a control **(G,H)** for 1 h, before first lactate was added for 1 h and then LPS **(E,G)** or Pam3Cys **(F,H)** was added for 24 h. **(A–D)** *n* = 6, **(E,F)** *n* = 4, **(G,H)** *n* = 3. *p < 0.05, Wilcoxon signed rank test.

To further decipher how lactate metabolism is influencing cytokines production, we treated PBMCs with the LDH-inhibitor oxamate. Oxamate increased LPS-induced, but decreased Pam3Cys-induced cytokine production (Figures 10A,B). Accordingly, the MCT-inhibitor α-CHCA increased LPS-stimulated IL-1β production, but decreased Pam3Cys-induced cytokine production (Figures 10C,D), again indicating differential roles for lactate metabolism in TLR4- vs. TLR2-stimulated cells. This phenotype was less pronounced in monocytes. Oxamate and α-CHCA both reduced LPS- and Pam3Cys-induced cytokine production (Figures 10E–H). Effects of the inhibitors in Pam3Cys-treated cells were more pronounced. Noticeably, effects of the inhibitors were similar in the presence and absence of extracellular lactate.

## Discussion

Extracellular lactate acutely shifts metabolism of human immune cells from glycolysis to oxidative phosphorylation *in vitro* and thereby influences cellular function. Whereas short-term lactate infusion *in vivo* has limited effects on *ex vivo* cytokine production, long-term exposure to lactate *ex vivo* has robust anti-inflammatory effects. This suggests rapid adaptations of human immune cells to lactate concentrations in the microenvironment, which may affect tissue-specific immune cell functions.

Studies investigating effects of lactate on inflammation have predominantly focused on GPR81-mediated effects, but recent studies suggest that lactate may also regulate inflammation by interfering with cellular metabolism (15, 16). Accordingly, we found that even low concentrations of lactate acutely modulate cellular metabolism of human immune cells. Although we detected acute changes in ECAR and OCR upon exposure of monocytes to lactate *in vitro*, this metabolic profile was not observed in monocytes isolated after lactate infusion *in vivo*. This may be a power issue, because only a limited number of patients could be selected for extracellular flux measurements. Alternatively, isolation procedures and incubation of cells in culture medium prior to the Seahorse run may have masked effects of lactate on cellular metabolism *in vivo*. Metabolic changes induced by lactate may be reversed rapidly upon exposure to a different environment due to metabolic flexibility of monocytes. This is supported by *in vitro* experiments demonstrating limited effects of a short-term lactate exposure on cytokine production (Figures 9C,D) and suggests that lactate may rather have immunomodulating effects in local microenvironments for instance in tumors or in the adipose tissue, where chronic elevation of lactate levels is more likely to occur than in the circulation. Duration of exposure to lactate may also decide on the outcome of the immunomodulating effects. Whereas several studies report anti-inflammatory effects of lactate after 24 h (11, 15), Samuvel et al. showed that cells pretreated with 20 mM lactate for 24 h followed by LPS in combination with lactate for another 24 h, had increased rather than decreased pro-inflammatory cytokine production (27). Consequently, timing effects, which might depend on intracellular signaling, possibly via GPR81, or on the ever-changing metabolic status of immune cells, should be carefully taken into account for future studies on *in vitro* or *in vivo* effects of lactate on immune cells.

Next to anti-inflammatory effects of lactate on murine macrophages, it has been shown that elevated levels of lactate can decrease IL-1β production of human PBMCs *in vitro* (11). Our results demonstrate that lactate also decreased the production of other pro-inflammatory cytokines (IL-6, TNFα). However, it appears that the immunomodulatory effect of lactate is stimulus-dependent. Whereas lactate clearly has anti-inflammatory effects in LPS-stimulated cells, effects are less pronounced in Pam3Cys-stimulated cells and IL-10 production even increased. This may in part be explained by the different metabolic profiles induced by LPS and Pam3Cys in human monocytes (20). Whereas LPS-stimulated monocytes rely more on glycolytic metabolism, Pam3Cys induces more oxidative phosphorylation, evidenced by increased levels of oxygen consumption as well as increased mitochondrial activity in Pam3Cys- vs. LPS-stimulated cells. Hence, the robust shift away from glycolysis toward oxidative phosphorylation induced by lactate may have a larger impact on the functional output of LPS-treated cells. Circulating lactate contributes to energy metabolism via the tricarboxylic acid (TCA) cycle (28). It is well established that LPS-stimulation of macrophages is disrupting the TCA cycle at citrate and succinate (29, 30). In contrast, the TCA cycle remains intact in Pam3Cys- vs. LPS-stimulated monocytes (20). Hence, this difference in TLR-specific metabolic rewiring may contribute to the differential effects of lactate on LPS- vs. Pam3Cys-stimulated cells.

Recently, it was reported that effector and regulatory T cells are differently equipped to function in low glucose, high lactate environments. Effector T cells have highly active glycolytic metabolism and their function is impaired by high lactate levels in the environment. Due to their active glycolysis, effector T cells require NAD^+^ regeneration, but high lactate levels promote the formation of pyruvate, thereby leading to LDH-mediated NAD^+^ depletion. In contrast, regulatory T cells can maintain their function in high lactate environments, due to upregulation of NAD^+^ regeneration via oxidative phosphorylation (31). Accordingly, differences in LDH-activity, as suggested by different lactate/pyruvate ratios in LPS- vs. Pam3Cys-stimulated monocytes (Figures 1D,E), and differences in use of oxidative phosphorylation may differentially regulate cellular functions in LPS- vs. Pam3Cys-stimulated cells exposed to high concentrations of lactate.

Our study points to an important role for LDH activity and LDH isoforms in the regulation of immune cell function. We observed that effects of extracellular lactate on metabolism were abolished when cells were treated with the general LDH-inhibitor oxamate (Figures 3A–D). Effects of oxamate on cytokine production however seemed to be independent of the presence of extracellular lactate. To pinpoint the role of LDHA vs. LDHB in immune cell function and further understand the contribution of extracellular vs. intracellular formed lactate on immune cell metabolism and cytokine release, studies with isoform-specific inhibition of LDH would be warranted. Research regarding lactate in immune cells has been focused on the LDH-mediated conversion of pyruvate into lactate, which leads to replenishment of NAD^+^ and secretion of lactate. However, LDH also catalyzes the conversion of lactate into pyruvate. Oxidation of lactate to pyruvate by LDHB is localized within mitochondria and supports lipid synthesis (32). It has been suggested that mitochondrial or intercellular lactate-shuttles are important for maintenance of metabolism, for example in the brain (33). Mitochondrial lactate oxidation in immune cells is rather unexplored, but our results suggest that lactate is used for oxidation in immune cells. LDHB activation in immune cells may favor the use of oxidative phosphorylation, which may have beneficial effects in some and detrimental in other situations.

For our *in vivo* study, we only analyzed effects of lactate infusions in patients with type 1 diabetes. Increased inflammasome activation and IL-1β production of PBMCs have been described in insulin-resistant patients with type 2 diabetes (34) and in newly diagnosed type 1 diabetes (26). Consequently, we expected lactate to have a pronounced anti-inflammatory effect in patients with T1D. In contrast, effects of the lactate infusion on metabolism and cytokine production were limited. This may be due to the fact that participants in our study were relatively young and well controlled, contributing to a low inflammatory status. Although a subgroup of patients had higher cytokine production at baseline compared with healthy controls, in general cytokine levels were in a similar range and we did not detect significant differences in blood leukocyte numbers between patients and controls. *Ex vivo* anti-inflammatory effects of lactate were similar in T1D patients compared with healthy individuals. It would be of interest for future studies to investigate effects of higher lactate concentrations or longer exposure times *in vivo*. Before we can investigate immunomodulating effects of lactate in more severe chronic inflammatory diseases (e.g., inflammatory bowel disease) or patients with acute inflammation (infections), we first need to better understand time-dependencies and mechanisms by which lactate influences human immune cells.

**Figure 11:**
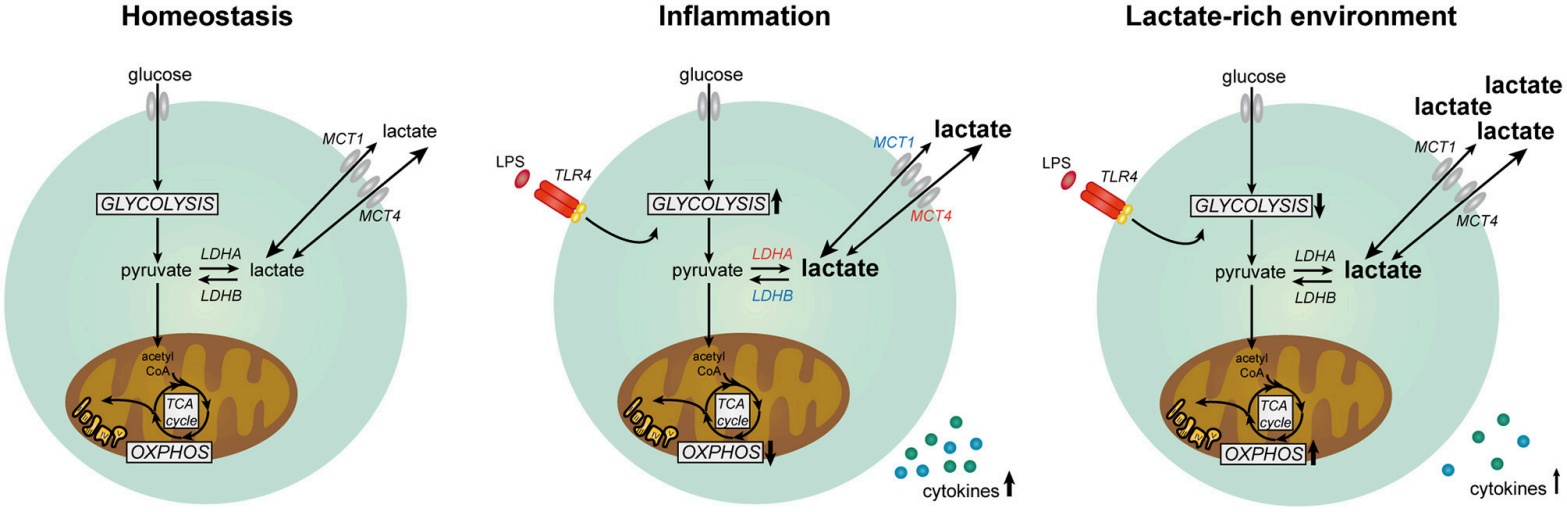
Lactate metabolism in human immune cells. Overview of the role of lactate in modulating metabolism and function in human immune cells. Expression of genes marked in red was upregulated and expression of genes marked in blue was downregulated in human immune cells upon stimulation with LPS.

In summary, our data suggest that lactate has anti-inflammatory effects on cytokine production of human PBMCs *in vitro* by modulating immune cell metabolism (Figure 11). Differential responses of LPS- and Pam3Cys-stimulated cells in high extracellular lactate conditions suggest that the effects of lactate are dependent on the metabolic signature of immune cells evoked by the stimulus. We propose that immunomodulatory effects of lactate may serve as a feedback signal to limit excessive inflammatory responses of highly glycolytic pro-inflammatory immune cells.

## Ethics statement

This study was carried out in accordance with the ethical principles taken from the Declaration of Helsinki and written informed consent was obtained from all study participants. The study was approved by the institutional review board of the Radboud University Medical Center. This study was part of a clinical trial registered at clinicaltrials.gov (NCT03286816).

## Author contributions

JR, HR, BdG, CT, and RS designed the study. JR planned and performed experiments and analyzed data. HR recruited patients, performed *in vivo* experiments and collected clinical data. GH and JR analyzed microarray data. AH performed gene expression analysis. JR and RS wrote the manuscript. All authors discussed the results and implications and commented on the manuscript.

### Conflict of interest statement

The authors declare that the research was conducted in the absence of any commercial or financial relationships that could be construed as a potential conflict of interest.

## Abbreviations

PBMCs: peripheral blood mononuclear cells
T1D: type 1 diabetes
LPS: lipopolysaccharide
MCT: monocarboxylate transporter
ECAR: extracellular acidification rate
OCR: oxygen consumption rate
LDH: lactate dehydrogenase
SRC: spare respiratory capacity.
